# Novel *exc* Genes Involved in Formation of the Tubular Excretory Canals of *Caenorhabditis elegans*

**DOI:** 10.1534/g3.119.200626

**Published:** 2019-03-18

**Authors:** Hikmat Al-Hashimi, Travis Chiarelli, Erik A. Lundquist, Matthew Buechner

**Affiliations:** Dept. of Molecular Biosciences, University of Kansas, Lawrence, KS, 66045, USA

**Keywords:** Tubulogenesis, lumen formation, endosomes, EXC-5, Charcot-Marie-Tooth Syndrome Type 4H

## Abstract

Regulation of luminal diameter is critical to the function of small single-celled tubes, of which the seamless tubular excretory canals of *Caenorhabditis elegans* provide a tractable genetic model. Mutations in several sets of genes exhibit the Exc phenotype, in which canal luminal growth is visibly altered. Here, a focused reverse genomic screen of genes highly expressed in the canals found 18 genes that significantly affect luminal outgrowth or diameter. These genes encode novel proteins as well as highly conserved proteins involved in processes including gene expression, cytoskeletal regulation, and vesicular and transmembrane transport. In addition, two genes act as suppressors on a pathway of conserved genes whose products mediate vesicle movement from early to recycling endosomes. The results provide new tools for understanding the integration of cytoplasmic structure and physiology in forming and maintaining the narrow diameter of single-cell tubules.

Tubule formation is an essential process during development of multicellular organisms, with the narrowest tubes occurring in structures as diverse as *Drosophila* trachea, floral pollen tubes, and mammalian capillaries (Lubarsky and Krasnow 2003; Sigurbjörnsdóttir *et al.* 2014). In *C. elegans*, the excretory system is comprised of cells that form single-celled tubules of three types: pore cells that wrap around a lumen to form a tube with an autocellular junction (“seamed tube”); a larger duct cell that forms a similar tube followed by dissolution of the junction to form a “seamless” tube; and the large excretory canal cell that extends four long seamless tubules (“canals”) throughout the length of the organism (Figure 1A-C) (Sundaram and Buechner 2016).

**Figure 1:**
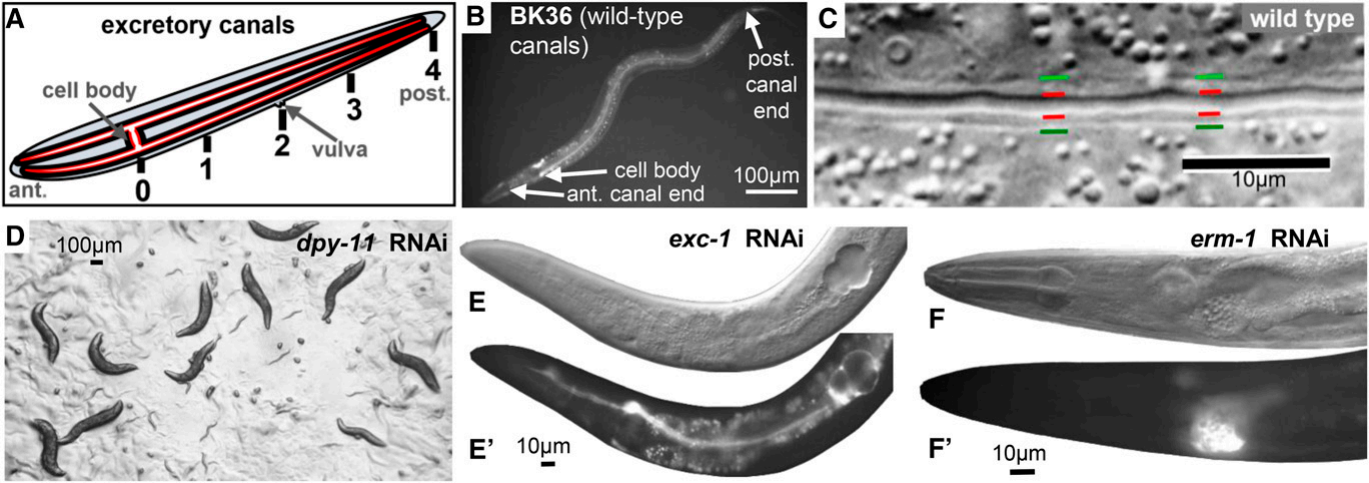
The excretory canals and induction controls. (A) Schematic diagram of the excretory canals extending over the full length of the worm with basal membrane (black) and apical membrane (red) surrounding a narrow lumen (white). Numbers 0-4 represent numerical assignments used to assess canal length. (B) Fluorescence image of L4 worm of strain BK36, showing GFP driven by the *vha-1* promoter in wild-type canals and other tissues (especially the head mesodermal cell on the dorsal side opposite the canal cell body). Focus shows the left-hand canal visible throughout length of body. Gut autofluorescence is apparent in center of body. (C) Magnified DIC image of excretory canal of wild-type worm (N2). Lines indicate boundaries of canal lumen/apical surface (red) and cytoplasmic/basal surface (green). (D-F) Controls to ensure strong induction of dsRNA synthesis for RNAi screen, in *rrf-3*(*pk1426*) animals expressing GFP in the canals: (D) Knockdown of cuticle collagen gene *dpy-11*. (E) DIC and (E’) GFP image of *exc-1* knockdown. (F) DIC and (F’) GFP image of *erm-1* knockdown.

Many mutants have been discovered that affect the length, guidance of outgrowth, or lumen diameter of the excretory canals. An initial set of such identified “*exc*” mutants, in which the canal lumen is abnormally wide, contains cysts, or is short, were mapped (Buechner *et al.* 1999), and found to include multiple alleles of some *exc* genes, but only single alleles of others. The frequency of mutations suggested that additional genes should have mutable excretory lumen effects. Studies by multiple laboratories indeed found alleles of other genes with Exc phenotypes (Khan *et al.* 2013; Kolotuev *et al.* 2013; Armenti *et al.* 2014; Lant *et al.* 2015; Gill *et al.* 2016; Forman-Rubinsky *et al.* 2017; Lant *et al.* 2018). Almost all of the original *exc* genes have now been cloned (Suzuki *et al.* 2001; Berry *et al.* 2003; Fujita *et al.* 2003; Praitis *et al.* 2005; Tong and Buechner 2008; Mattingly and Buechner 2011; Shaye and Greenwald 2015; Grussendorf *et al.* 2016; Al-Hashimi *et al.* 2018), and found to affect multiple well-conserved cell processes, including ion transport, formation of cytoskeletal structures, and vesicle recycling pathways. The initial screen sought primarily non-lethal genetic effects, but several of the subsequently identified genes were lethal when null.

RNAi studies have been particularly useful in determining roles of excretory canal genes where the null allele is lethal, such as the gene encoding the NHR-31 nuclear hormone receptor (Hahn-windgassen and Van gilst 2009), the ABI-1 Abelson-Interactor (Mcshea *et al.* 2013), and the PROS-1 transcription factor (Kolotuev *et al.* 2013). In addition, null mutations in genes that affect the patency of the neighboring excretory duct cell (*e.g.*, LET-4 (Mancuso *et al.* 2012) and LPR-1 (Forman-Rubinsky *et al.* 2017)) are lethal.

In order to identify other genes affecting the process of tubulogenesis and tubule maintenance in the excretory canals, we undertook a targeted genomic RNAi screen to identify excretory canal genes that exhibit lumen alterations (“Exc” phenotypes) when knocked down. This screen confirmed or identified 18 genes preferentially expressed in the canals that showed effects on lumen and/or outgrowth of the excretory canals, including 10 genes with no prior known phenotypic effects on the canals. In addition, two knockdowns suppressed effects of mutation of the *exc-5* guanine exchange factor gene affecting canal endosomal recycling, and therefore represent potential regulators of vesicle transport needed for single-cell tubulogenesis.

## Materials and Methods

### Nematode genetics

*C. elegans* strains (Table 1) were grown by use of standard culture techniques on lawns of *Escherichia coli* strain BK16 (a streptomycin-resistant derivative of strain OP50) on nematode growth medium (NGM) plates (Sulston and Hodgkin 1988). All strains were grown and evaluated for canal phenotypes at 20°. Worms observed in this study were young adults or adults.

**Table 1:** List of strains used in this study, with genotype descriptions

STRAIN	GENOTYPE	DESCRIPTION	REFERENCE
BK36	*unc-119(ed3)* III; *qpIs11* [*unc-119*; *P_vha-1_*::*gfp*] I	N2 with integrated GFP marker expressed in excretory canal cytoplasm	(Mattingly and Buechner 2011)
BK540	*rrf-3*(*pk1426*) II; *qpIs11* [*unc-119*; *P_vha-1_*::*gfp*] I	RNAi-sensitized strain expressing GFP in canals	This study
BK541	*sid-1(pk3321)* II; *qpIs11* [*unc-119*; *P_vha-1_*::*gfp*] I	Systemic RNAi-impaired strain expressing GFP in canals	This study
BK542	*exc-2*(*rh90*) X; *qpIs11* [*unc-119*; *P_vha-1_*::*gfp*] I	*exc-2*(*rh90*) expressing GFP in canals	This study
BK543	*exc-3*(*rh207*) X; *qpIs11* [*unc-119*; *P_vha-1_*::*gfp*] I	*exc-3*(*rh207*) expressing GFP in canals	This study
BK544	*exc-4*(*rh133*) *qpIs11* [*unc-119*; *P_vha-1_*::*gfp*] I	*exc-4*(*rh133*) expressing GFP in canals	This study
BK545	*exc-5*(*rh232*) IV; *qpIs11* [*unc-119*; *P_vha-1_*::*gfp*] I	*exc-5*(*rh232*) expressing GFP in canals	This study
BK546	*exc-7*(*rh252*) II; *qpIs11* [*unc-119*; *P_vha-1_*::*gfp*] I	*exc-7*(*rh252*) expressing GFP in canals	This study
BK547	BK540; *exc-2*(*rh90*) X	*exc-2*(*rh90*) expressing GFP in canals in RNAi-sensitized background	This study
BK548	BK540; *exc-3*(*rh207*) X	*exc-3*(*rh207*) expressing GFP in canals in RNAi-sensitized background	This study
BK549	BK540; *exc-4*(*rh133*) I	*exc-4*(*rh133*) expressing GFP in canals in RNAi-sensitized background	This study
BK550	BK540; *exc-5*(*rh232*) IV	*exc-5*(*rh232*) expressing GFP in canals in RNAi-sensitized background	This study
VC20239	*exc-15*(E89K) mutation	“million mutation” strain homozygous at ∼3-6 loci	(Thompson *et al.* 2013)
VC20363	H09G03.1(P15S) mutation	“million mutation” strain homozygous at ∼3-6 loci	(Thompson *et al.* 2013)
VC20573	H09G03.1(G67R) mutation	“million mutation” strain homozygous at ∼3-6 loci	(Thompson *et al.* 2013)
VC40373	*exc-13*(C44Y) mutation	“million mutation” strain homozygous at ∼3-6 loci	(Thompson *et al.* 2013)
VC40556	T19D12.9(Q61X) mutation	“million mutation” strain homozygous at ∼3-6 loci	(Thompson *et al.* 2013)
VC40788	C09F12.3(P41S) mutation	“million mutation” strain - Died at thaw; not used	(Thompson *et al.* 2013)

Each nematode strain (wild-type N2, and *exc-2*, *exc-3*, *exc-4*, *exc-5*, and *exc-7*) was crossed to strain BK36, which harbors a chromosomal insertion of a canal-specific promoter driving cytoplasmic GFP expression (P*_vha-1_*::*gfp*). Strains were then sensitized for RNAi treatment by crossing them to mutant strain BK540 (a strain carrying *rrf-3*(*pk1426*) in addition to the same chromosomal *gfp* insertion as above) and selecting in the F2 generation for homozygous *rrf-3* deletion allele and appropriate *exc* mutation. (As *exc-7* maps very close to *rrf-3*, the *exc-7* strain carrying *gfp* was not crossed to BK540 and was not sensitized to RNAi). For all sensitized strains, the *rrf-3* deletion was confi rmed via PCR using the forward primer ^5′^TGCTTTGGATATTGCCGAGCAC^3′^, reverse primer ^5′^GGAGATCTCCGAGCCCTAGAC^3′^, and a reverse nested primer ^5′^CATCGCCAGGCCAACTCAATAC^3′^. As a negative control, we crossed BK36 to RNAi-refractive strain NL3321 *sid-1*(*pk3321*).

### RNAi Screen

The Ahringer RNAi bacterial library (Kamath *et al.* 2003) was utilized for this study. Bacterial clones used are listed in Supp. Tables S1 and S2, available on Figshare. Overnight cultures were prepared by inoculating bacteria in 5 ml LB + ampicillin (100µg/ml) + tetracycline (12.5µg/ml), and cultured at 37° for 16 hr. In order to induce the bacteria with IPTG, overnight cultures were moved to fresh media, incubated at 37° with rotation until cultures reached an O.D._600_ in a range from 0.5 to 0.8. IPTG was then added to the culture to a final concentration of 95μg/ml along with ampicillin at 100μg/ml. The cultures were then incubated with rotation at 37° for ninety minutes followed by re-induction with IPTG and ampicillin, and another ninety minutes of incubation at 37° with rotation. Finally, IPTG and ampicillin were added one last time just prior to using these bacteria to seed NGM in 12-well plates and Petri dishes. Plates were then incubated at room temperature for 24 hr in order to dry. 3-4 L2 worms were added to the plates, and their F1 young adult progeny were evaluated for phenotypes in the excretory canals. Each set of genes tested was induced together with induction of the *sid-1* negative control strain BK541 and of two positive control strains: a plate of bacteria induced to knock down *dpy-11* (which affects the hypoderm but not the canals) (Brenner 1974) (Figure 1D); a plate of bacteria induced to knock down *exc-1*, (which affects predominantly the canals and amphid sheaths but does not substantially affect viability) (Grussendorf *et al.* 2016) (Figure 1E); and a plate of bacteria induced to knock down *erm-1* (Figure 1F), which causes severe defects in excretory canal length and lumen diameter, as well as similar lethal defects in the intestine (Khan *et al.* 2013), as a test that the RNAi induced partial as well as full knockouts. Induction was considered successful and plates were screened only if worms grown on the control plates showed the appropriate phenotypes in at least 80% of the surviving progeny.

For each tested gene, the induced bacteria were seeded on one 12-well plate and one 60mm plate. Two or three L2 nematodes were placed on the bacterial lawn of each well, and screened for phenotypes in the 4^th^, 5^th^, and 6^th^ days of induction. Each gene was tested via RNAi treatment of twelve different strains of worms, shown in Table 1, while the sole 60mm plate was used for further analysis of animals with wild-type canals (strain BK540, Table 1) grown on the RNAi-expressing bacteria. For assessment of a canal effect, a minimum of five young adult animals (out of 100-150 young adult animals examined on the 4^th^-6^th^ day after induction; some knockdowns affected growth, so number of animals examined was lower) showing a canal phenotype were collected, examined, and in most cases photographed; for most genes, 10-20 affected animals were examined closely.

For the genes showing effects, the entire experiment was subsequently repeated at least once, with induction and growth of bacteria solely on 60mm plates and feeding tested on BK540 (RNAi-sensitized wild-type with integrated canal marker) worms; numerical results of the young adult animals in these later tests are reported in Table 2. Finally, we sequenced PCR-amplified inserts of the bacterial RNAi-inducing plasmids for nine of these clones corresponding to previously undesignated genes in order to confirm that there was no cross-contamination in our work or between Ahringer clones (Qu *et al.* 2011). For the other clones, we noted that RNAi also caused the expected phenotypes associated with the named genes. While it was not possible to exclude the possibility that a small contaminant was the source of the RNAi response, the vast majority of bacteria fed to the treated worms was of the appropriate clone.

**Table 2:** Ahringer clones causing RNAi effects on GFP-labeled canal length in RNAi-sensitized animals

Gene	Nematode Gene Affected	Most Frequent Canal RNAi Phenotype	# canals examined in later tests	% mutant canals	Avg. mutant canal length	Std. Dev. of mutants	*p*-value *vs.*wild-type
Strain BK540		Control: wild-type canals	100	0%	4.0	±0.0	—
Knockdowns exhibiting effects with high statistical confidence:							
ceh-6*	K02B12.1	large fluid-filled cysts	33	100%	1.1	±0.35	6.7x10^−24^
*exc-10*	T25C8.1	large fluid-filled cysts	63	68%	3.3	±0.39	2.7x10^−24^
*egal-1*	C10G6.1	medium-sized fluid-filled cysts	40	100%	1.1	±0.37	5.7x10^−36^
mop-25.2*	Y53C12A.4	medium-sized fluid-filled cysts, vesicles along swollen cytoplasm	20	100%	1.1	±0.22	3.4x10^−23^
*exc-11*	F41E7.1	medium-sized fluid-filled cysts	34	97%	1.7	±0.52	1.4x10^−30^
*exc-12*	T05D4.3	medium-sized fluid-filled cysts	53	79%	2.6	±0.59	9.4x10^−28^
*best-3*	C01B12.3	swollen luminal tip	36	28%	3.4	±0.31	5.8x10^−7^
gck-3*	Y59A8B.23	swollen tip, vesicles, convolutions	76	57%	3.5	±0.26	1.6x10^−20^
*fbxa-183*	F44E7.6	swollen tip with vesicles	125	66%	3.1	±0.27	3.0x10^−30^
*exc-14*	K11D12.9	vesicles along swollen cytoplasm	36	100%	1.4	±0.47	9.5x10^−34^
*exc-15*	T08H10.1	vesicles along swollen cytoplasm	41	27%	3.5	±0.15	4.3x10^−7^
*exc-13*	C03G6.5	vesicles along swollen cytoplasm	79	19%	3.5	±0.32	1.2x10^−6^
cyk-1*	F11H8.4	vesicles along swollen cytoplasm	77	77%	3.1	±0.28	2.8x10^−34^
inx-12*	ZK770.3	periodic cytoplasmic beads	46	96%	2.7	±0.50	2.2x10^−35^
inx-13*	Y8G1A.2	periodic cytoplasmic beads	50	80%	2.8	±0.51	3.3x10^−29^
ceh-37*	C37E2.5	periodic cytoplasmic beads	40	30%	3.3	±0.31	7.7x10^−8^
*mxt-1*	Y18D10A.8	cytoplasmic beads with vesicles	59	93%	2.6	±0.51	2.0x10^−38^
vha-5*	F35H10.4	beads, vesicles, swollen cytoplasm	78	59%	2.5	±0.79	7.7x10^−22^
Knockdowns exhibiting effects with lower statistical confidence:							
*dhhc-2*	Y47H9C.2	cytoplasmic beads with vesicles	57	21%	3.5	±0.21	2.3x10^−6^
—	T19D12.9	swollen tip, vesicles, convolutions	83	26%	3.3	±0.47	1.3x10^−6^
*gst-28*	Y53F4B.31	swollen tip with vesicles	26	27%	3.4	±0.38	7.8x10^−6^
*gsr-1*	C46F11.2	swollen tip, vesicles, convolutions	73	5%	3.4	±0.30	0.030
—	H09G03.1	vesicles along swollen cytoplasm	179	7%	3.3	±0.46	4.7x10^−4^
—	C09F12.3	vesicles along swollen cytoplasm	88	8%	3.3	±0.22	4.3x10^−3^
*exc-5 (qp110)*		large cysts at canal tips	118	100%	1.35	±1.35	
Knockdowns exhibiting suppression effects on *exc-5*:							*p*-value *vs. exc-5*
*suex-1*	F12A10.7	suppresses *exc-5* mutant cysts	224	95%	2.25	±0.65	1.3x10^−18^
*suex-2*	C53B4.1	suppresses *exc-5* mutant cysts	207	97%	2.26	±0.73	1.7x10^−14^

**Figure 2:**
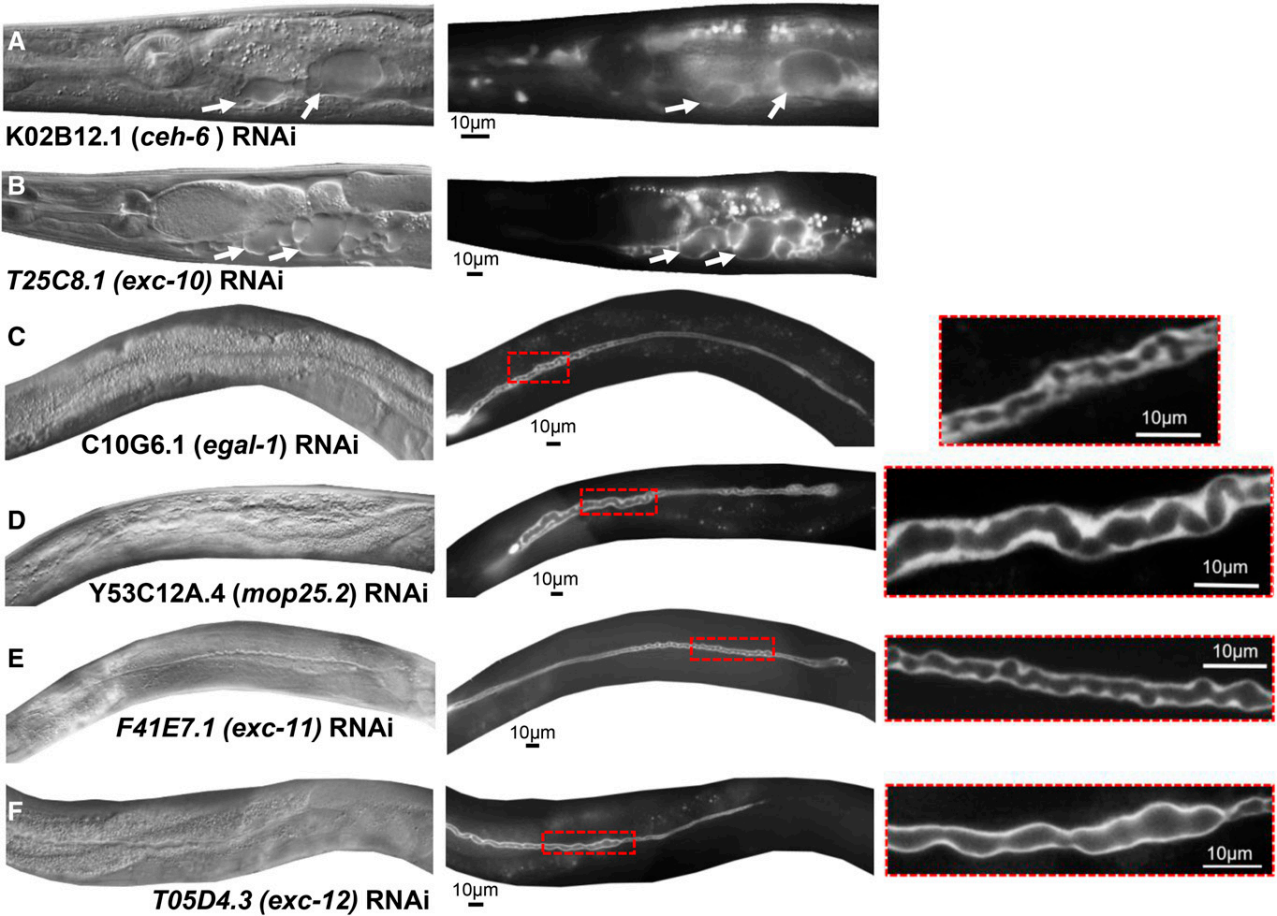
RNAi knockdowns causing formation of fluid-filled cysts or swollen lumen. DIC images (left) and GFP fluorescence (center) of representative animals exhibiting RNAi-knockdown phenotypes: (A) *ceh-6*; (B) *T25C8.1* (*exc-10*); (C) *egal-1*; (D) *mop-25.2*; (E) *F41E7.1* (*exc-11*); (F) *T05D4.3* (*exc-12*). Panels on right are enlargements of areas outlined in red in center panels. Arrows: Representative medium and large fluid-filled cysts. All bars, 10 µm.

**Figure 3:**
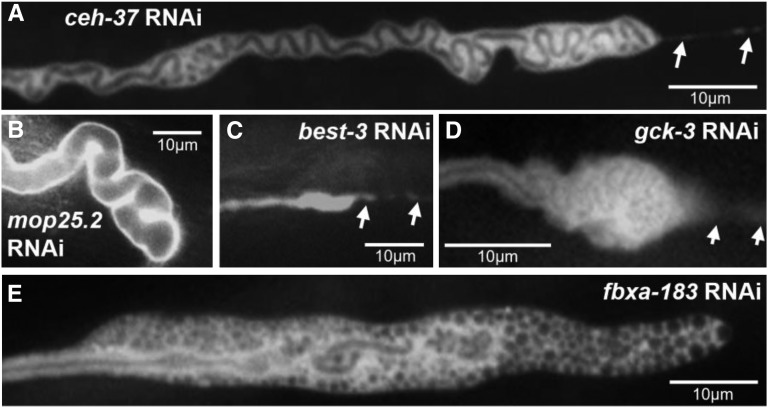
RNAi knockdowns causing swelling at end of lumen. GFP fluorescence images of swollen canals at termination of lumen caused by RNAi knockdown of genes (A) *ceh-37*; (B) *mop-25.2*; (C) *best-3*; (D) *gck-3*; (E) *fbxa-183*; all images show regions of convoluted canals. Some additional areas in panels D and E appear as individual separated small cysts or large vesicles. Arrows: Cytoplasmic tail continuing past termination of lumen in panels A, C, and D.

**Figure 4:**
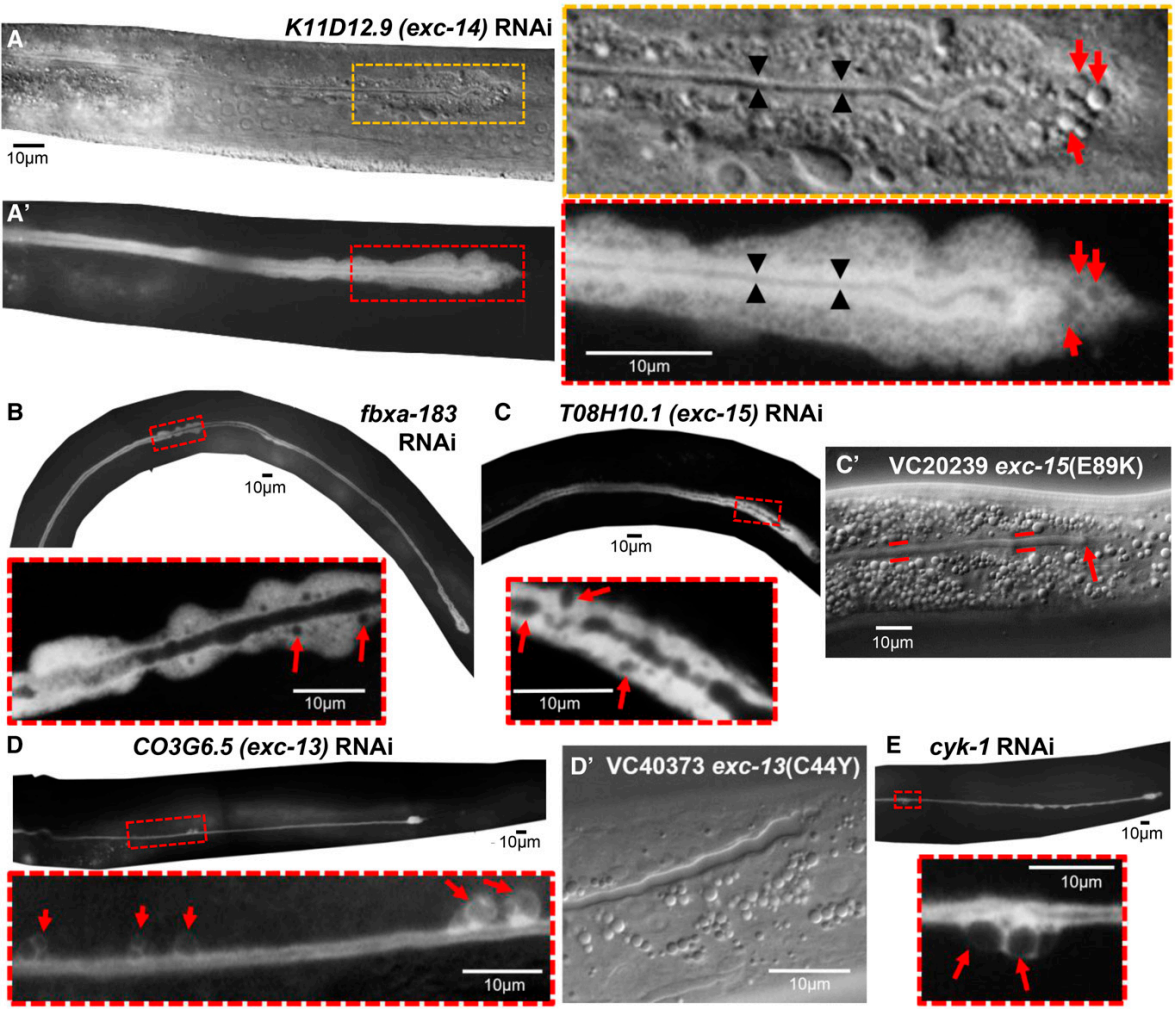
RNAi knockdowns causing vesicle accumulations and irregular basal membrane along canal length. (A) DIC and (A’) GFP fluorescence images of distal tip of canal of representative animal knocked down for *K11D12.9* (*exc-14*). Boxed areas are enlarged to right. Thin lumen indicated by black arrowheads is surrounded by area of bright GFP fluorescence. Distorted cytoplasmic shape is filled with large number of vesicles (red arrows). (B-E) GFP fluorescence of representative animals knocked down for genes: (B) *fbxa-183*; (C) *T08H10.1 (exc-15)*; (D) *C03G6.5 (exc-13)*; (E) *cyk-1*. Boxed areas enlarged below each panel show areas along the canals where cytoplasm surface is swollen with vesicles, and basal surface is irregular and noticeably wider than in wild-type animals. Arrows show enlarged vesicles or cysts. (C’, D’) DIC photographs of canals in “million mutation” strains containing mutations in genes *exc-15* and *exc-13*. Red lines in Fig. C’ show wider than normal canal terminating abruptly.

**Figure 5:**
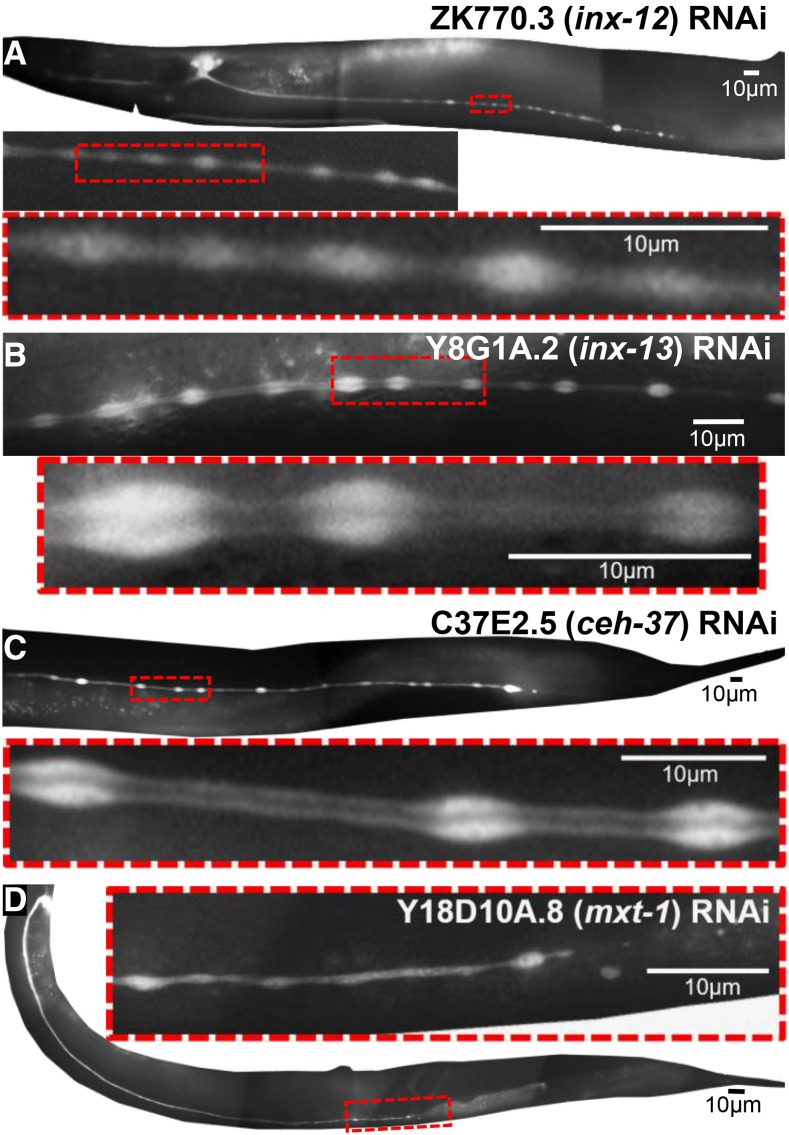
RNAi knockdowns causing periodic cytoplasmic swellings. GFP fluorescence images of swellings (“beads”) along length of canals. Boxed insets of marked areas are magnified to show width of lumen in regions within and between beads. RNAi knockdown of: (A) *inx-12*; (B) *inx-13*; (C) *ceh-37*; (D) *mxt-1*.

**Figure 6:**
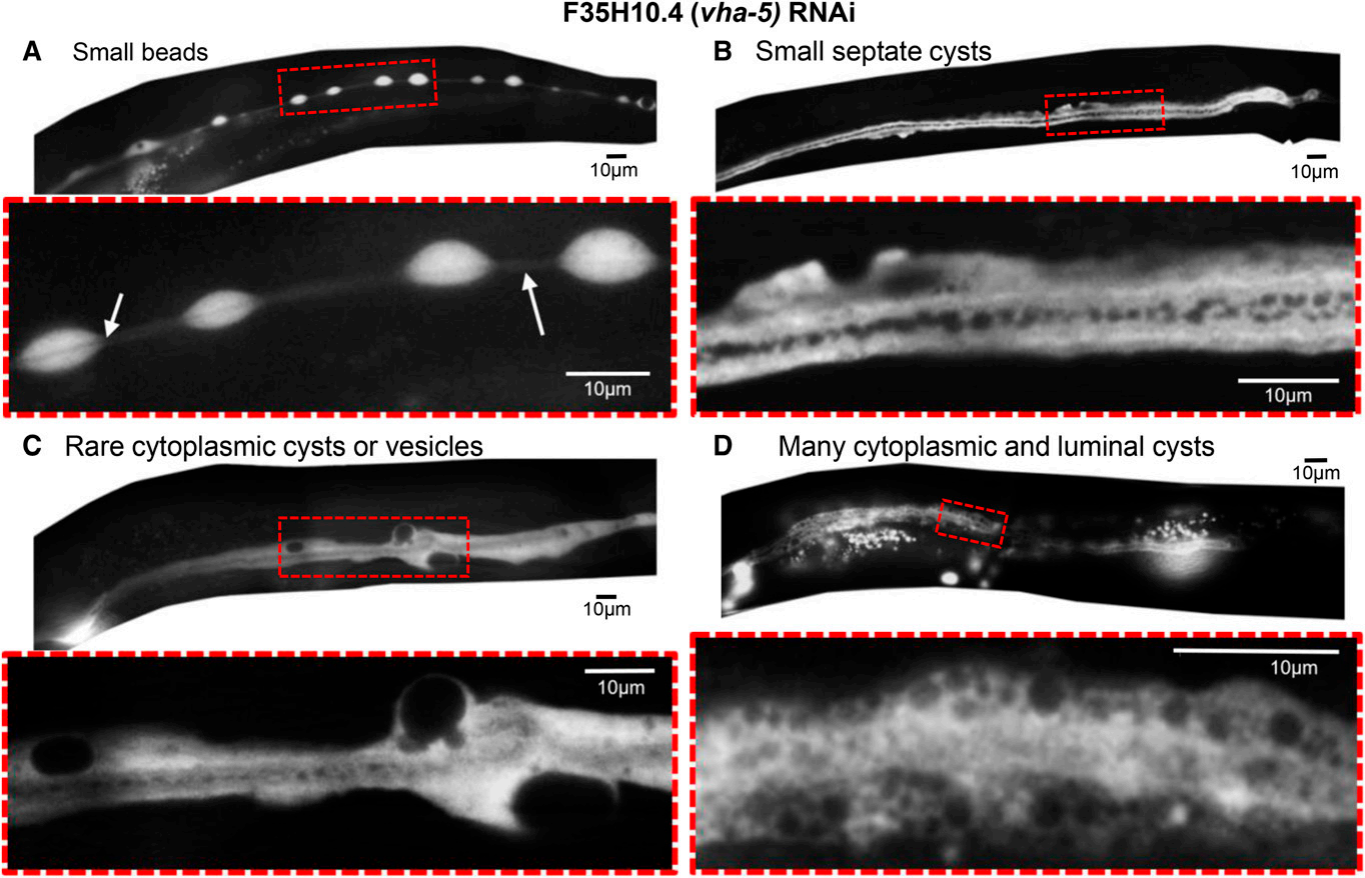
Knockdown of *vha-5* leads to a wide range of phenotypes. (A-D) GFP fluorescence of four different worms exhibiting a range of excretory canal phenotypic severity in response to *vha-5* knockdown. For each animal, the area boxed in red is enlarged below. (A) Periodic cytoplasmic swellings along lumen of canal. Arrows show visible lumen of normal diameter. (B) Small septate cysts in the lumen of the canal, surrounded by area of bright GFP fluorescence, and somewhat irregular diameter cytoplasm. (C) Lumen with septate cysts similar to 4B and surrounded by cytoplasm of more irregular diameter containing large cysts/vesicles. (D) Wider-diameter lumen with larger cysts, surrounded by cytoplasm filled with vesicles in a wide range of sizes.

Knockouts of genes found to have effects on *C. elegans* excretory canals. Thick lines separate knockdowns that showed effects with high confidence from knockdowns with effects of lower confidence, and knockdowns that exerted suppression effects on *exc-5* mutants. Thin lines separate genes by the most common phenotype exhibited in knockdowns, and correspond to panels in Figures 2, 3, 4, 5, and 6, respectively. Asterisks indicate genes previously demonstrated to have effects on the excretory canals. *p*-value was determined via 3x2 Fisher’s exact test (see Materials & Methods), with 1x10^−6^ viewed as determining high significance (*exc-13* was included as being close to this threshold, plus showing effects in “million mutation” animals).

Finally, the bacterial construct from the Ahringer Library for knockdown of F12A10.7, the *suex-1* gene, also included a small number of base pairs of the nearby gene F12A10.1. The identity of the suppressing gene was confirmed through microinjection of synthesized dsRNA specific to the F12A10.7 transcript into the gonad of the *exc-5* null mutant strain BK545, which caused the appearance of progeny with canals of wild-type length.

### Microscopy

Living worms were mounted on 3% agarose pads to which were added 0.1μm-diameter Polybead polystyrene beads (Polysciences, Warrington, PA) to immobilize the animals (Kim *et al.* 2013). Images were captured with a MagnaFire Camera (Optronics) on a Zeiss Axioskop microscope equipped with Nomarski optics and fluorescence set to 488nm excitation and 520nm emission. Adobe Photoshop software was used to combine images from multiple sections of individual worms and to crop them. Contrast on images was uniformly increased to show the excretory canal tissue more clearly.

### Canal Measurements

Effects on excretory canal length were measured and analyzed as described (Tong and Buechner 2008). Canal length was scored by eye on a scale from 0.0-4.0: A score of (4.0) was given if the canals had grown out to full length; canals that extended halfway past the vulva (midbody) were scored as (3.0); at the vulva (2.0); canals that ended halfway between the cell body and the vulva were scored as (1.0); and if the canal did not extend past the cell body, the canal was scored as (0.0). Lengths between these waypoints were visually estimated. For knockdowns where fluid-filled cysts were evident, cyst size was rated as large (cyst diameter at least half the width of the animal), medium (one-quarter to one-half animal width), or small (up to one-quarter animal width).

For statistical analyses, canals were binned into three categories for length (scores 0-1, scores 1.5-3.8, and score 3.9-4), and the results analyzed via a 3x2 Fisher’s Exact Test (www.vassarstats.net). A *p*-value at or below 10^−6^ was regarded as strong statistical significance that disruption of the gene caused an excretory canal defect, as per earlier studies on the canals (Tong and Buechner 2008).

Even with multiple observations, knockdown of five putative novel *exc* genes yielded a lower statistical confidence in the ability of RNAi knockdown to affect canal structure (Tables S1 and S2, available on FigShare). For these genes, “million mutation” strains (Thompson *et al.* 2013) containing homozygous point mutations in these genes were requested from the *Caenorhabditis* Genetics Center (Minneapolis, MN), and canals of young adults observed (Table S2). The selected strains contain mutations causing missense or nonsense mutations in the desired genes (as well as similar mutations in a few other genes). Observations of canal defects in two of these strains supported the conclusions made from finding canal defects in knockdowns for two of the genes, now named *exc-13* and *exc-15*, but still provided ambiguous results for knockdowns of two other genes (Table S2). *exc-13* is therefore listed as a novel *exc* gene, even though its statistical significance (*P* = 1.2x10^−6^) is not quite at the cutoff of 1.0x10^−6^ listed above. For a fifth gene, C09F12.3, the “million mutation strain” VC40788 showed substantial embryonic lethality and could not be supplied by the *Caenorhabditis* Genetics Center, which reported little obvious evidence of gross defects such as edema from lack of canal function within the dead animals. Photographs of affected canals of strains with only moderate statistical support for canal effects are provided in Supplemental Figure S1.

### Reagent and Data Availability

All nematode strains used in this study are listed in Table 1. Bacterial clone numbers tested, and summary of test results are presented in Tables S1 and S2, available on Figshare. Gene names *exc-10* through *exc-15* and *suex-1* and *suex-2* have been registered with Wormbase (www.wormbase.org). Sensitized *exc* mutant strains are available upon request, and may be made available through the Caenorhabditis Genetics Center (CGC), University of Minnesota (cgc.umn.edu), pending acceptance to that repository. Other strains are available upon request. Supplemental material available at Figshare: https://doi.org/10.25387/g3.7710362.

## Results

### A focused RNAi screen for new exc mutations

A study of genomic expression in *C. elegans* was previously undertaken by the Miller lab (Spencer *et al.* 2011). In that study, lists of genes highly expressed in various tissues, including 250 genes preferentially expressed in the excretory canal cell, were made public on the website WormViz (http://www.vanderbilt.edu/wormdoc/wormmap/WormViz.html). Of the corresponding strains in the Ahringer library of bacteria expressing dsRNA to specific *C. elegans* genes (Kamath *et al.* 2003), 216 grew well, and were tested for effects on the various *C. elegans* strains (Table S1, S2).

The excretory canal cell has some characteristics similar to those of neurons: long processes guided by netrins and other neural guidance cues (Hedgecock *et al.* 1987), as well as early expression of the gene EXC-7/HuR/ELAV (Fujita *et al.* 2003), and so was considered potentially refractory to feeding RNAi (Calixto *et al.* 2010). We crossed strain BK36 (Figure 1B), containing a strong canal-specific integrated *gfp* marker, to a mutant in the *rrf-3* gene (*pk1426*) in order to increase sensitivity to RNAi (Simmer *et al.* 2002) to create strain BK540. In addition, we also crossed the same *gfp* marker and *rrf-3* mutation to excretory canal mutants *exc-2*, *exc-3*, *exc-4*, *exc-5*, and *exc-7* (except that *exc-7* was not RNAi-sensitized; see Materials and Methods). This was done in order to determine if the tested gene knockdowns interacted with known *exc* genes affecting excretory canal tubulogenesis, since double mutants in some *exc* genes (*e.g.*, *exc-3*; *exc-7* double mutants (Buechner *et al.* 1999) exhibit more severe canal phenotypes than either mutant alone.

We demonstrated the effectiveness of the treatment by performing successful knockdowns of canal-specific and -non-specific genes in these strains. Control knockdowns of *dpy-11* resulted in short worms with normal canal phenotypes (Figure 1D), while knockdown of *exc-1* caused formation of variable-sized cysts in a shortened excretory canal, with no other obvious phenotypes (Figure 1E). Knockdown of the ezrin-moesin-radixin homolog gene *erm-1* (Göbel *et al.* 2004; Khan *et al.* 2013) also caused severe malformation of the canals visible in 80% of surviving treated worms (Figure 1F). A deletion mutant of this gene is often lethal due to cystic malformation of the intestine as well as the canals (Göbel *et al.* 2004), while our treatment allowed many animals to survive to adulthood and reproduce. This result is consistent with our RNAi treatment causing variable levels of gene knockdown (Timmons and Fire 1998) in the excretory canals.

Of the 212 non-control genes tested, 182 caused no obvious phenotypic changes to the canals of BK540 worms, and 4 gave very low numbers (less than 5) of animals with mild defects (Supp. Tables S1, S2). Knockdown of 24 genes caused noticeable defects in the development of the excretory canals in at least 5 worms. These genes were subsequently retested via feeding RNAi at least once, which confirmed an RNAi effect for 18 of the tested strains (Table 2), with statistically lower frequency of effect observed for knockdowns of the other 6 genes (Supp. Table S2, Supp. Fig. S1). Canal length was rated according to a measure shown in Figure 1A, in which lack of extension past the excretory cell body was rated 0, extension to the animal midbody marked by the position of the vulva was measured as 2, and full extension was rated as 4. The average canal length of affected animals was characteristic for the gene knocked down (Table 2).

For this RNAi screen, several caveats apply to the reported results. The effects of feeding RNAi can be highly variable based on the strength of induction of bacterial transcription (Hull and Timmons 2004). This use of feeding RNAi knockdown, however, does allow observation of gene effects where knockouts have been reported to be lethal. For example, null mutations in the canal-expressed gene *ceh-6* are lethal due in part to loss of expression of a wide range of channels and transporters both in the canals and in the rectal epithelium (Burglin and Ruvkun 2001; Armstrong and Chamberlin 2010). In the present screen, however, we found many viable *ceh-6*-knockdown animals, 100% of which contained strong luminal defects in the canals, and this gene was therefore used as a positive control for assessing whether RNAi knockdown occurred in the excretory canals. A corollary of this variability is that the statistical results reported here represent a minimum effect upon canal morphology caused by knockdown. Each gene noted (Tables 2 and 3) represents the results of an initial test where at least five viable animals display shortened canal lumens, followed by further tests where the number of multiple affected canals are reported in young adults. The length of the lumen in wild-type young adult canals is highly invariable (4.0 +/− 0.0), so even small numbers of shortened canals can represent a significant effect upon lumen formation. Finally, other cells and tissues affect canal outgrowth and morphology; mutations in basement membrane proteins affect canal length and direction of outgrowth (Hedgecock *et al.* 1987; Schmidt *et al.* 2009; Mcshea *et al.* 2013), while mutations affecting patency of the neighboring excretory duct cells can also cause excretory edema (Mancuso *et al.* 2012; Gill *et al.* 2016; Forman-Rubinsky *et al.* 2017). The mutations reported here show an Exc phenotype, and all genes are highly expressed within the excretory canal cell, but further study of each gene and its product is needed to determine the time of action and to confirm the location of action within the excretory cell.

**Table 3:** Identity of tested genes and encoded proteins that affect excretory canal morphology

		Protein Class		
Gene	Clone	Short Description of Known or Inferred Protein Function	References
		Transcriptional and post-transcriptional factors		
ceh-6*	K02B12.1	homeobox transcription factor	(Burglin and Ruvkun 2001; Armstrong and Chamberlin 2010)
ceh-37*	C37E2.5	Otx homeobox transcription factor	(Lanjuin *et al.* 2003; Hench *et al.* 2015)
*egal-1*	C10G6.1	Egalitarian exonuclease, regulates dynein	(Kipreos and Pagano 2000)
*fbxa-183*	F44E7.6	F-box protein, possible effects on RNA	(Fridolfsson *et al.* 2010)
*exc-14*	K11D12.9	RING finger, possible E3 ubiquitin ligase	(Kaneko *et al.* 2016)
*mxt-1*	Y18D10A.8	translation regulation	(Peter *et al.* 2015)
		Cytoskeletal proteins and regulators		
cyk-1*	F11H8.4	*Diaphanous* formin	(Severson *et al.* 2002; Shaye and Greenwald 2016)
		Transporters, channels, and receptors		
inx-12*	ZK770.3	innexin gap junction protein	(Altun *et al.* 2009; Hall 2017)
inx-13*	Y8G1A.2	innexin gap junction protein	(Altun *et al.* 2009; Hall 2017)
vha-5*	F35H10.4	vacuolar ATPase component	(Oka *et al.* 2001; Liégeois *et al.* 2006; Hahn-Windgassen and Van Gilst 2009)
*best-3*	C01B12.3	bestrophin chloride channel	(Strauß *et al.* 2014)
*exc-11*	F41E7.1	Na^+^/H^+^ solute carrier (SLC9 family)	(Fuster and Alexander 2014)
		Vesicle movement regulators		
gck-3*	Y59A8B.23	germinal center WNK kinase protein	(Falin *et al.* 2009; Gagnon and Delpire 2012)
mop-25.2*	Y53C12A.4	scaffolding for endocytic recycling	(Lant *et al.* 2018; Sun *et al.* 2018)
		Enzymatic activities		
*exc-10*	T25C8.1	sedoheptulose kinase	(Phornphutkul *et al.* 2001; Wamelink *et al.* 2008)
*exc-15*	T08H10.1	aldo-keto reductase	(Barski *et al.* 2008)
		Unknown function		
*exc-12*	T05D4.3	Nematode-only transmembrane protein	
*exc-13*	C03G6.5	Nematode conserved-domain protein	
*suex-1*	F12A10.7	*Caenorhabditis*-only glycine-rich protein	
*suex-2*	C53B4.1	solute carrier (SLC22 family)	(Cram *et al.* 2006; Nigam 2018)

Protein class shows known function, or function of closest homologs, of products of genes where knockouts affected morphology on *C. elegans* excretory canals. Thick line separates knockdowns that caused defects in canal morphology, and knockdowns that suppressed effects of *exc-5* mutation. Thin lines separate genes by class of protein encoded. Asterisks indicate genes previously demonstrated to have effects on the excretory canals.

### Excretory Canal Phenotypes

In knockdown animals, the posterior canals did not extend fully to the back of the animal (Table 2, Figure 1E, 1F). The length of the canal lumen was often the same as the length of the canal cytoplasm, but in many cases the visible lumen (seen as a dark area in the center of the GFP-labeled cytoplasm) was substantially shorter than the length of the canal cytoplasm.

In addition to effects on canal length, the shape and width of the canal lumen and/or canal cytoplasm was affected by specific gene knockdown. We present knockdown results according to the most-frequently observed phenotype seen for specific gene knockdowns, but knockdown of a gene often yielded different phenotypes in different individuals.

#### Cystic canals:

Two gene knockdowns, of *ceh-6* and of T25C8.1 (which will be referred to as *exc-10*), primarily resulted in the formation of large fluid-filled cysts (Figure 2A, 2B), similar to those seen in *exc-2*, *exc-4*, and *exc-9* mutants (encoding an intermediate filament, a CLC chloride channel, and a LIM-domain protein involved in vesicle-trafficking (Berry *et al.* 2003; Tong and Buechner 2008; Al-Hashimi *et al.* 2018)). The homeobox gene *ceh-6* encodes a well-studied POU-domain transcription factor that defines expression of many genes in the canal and rectal epithelium, and is regarded as a master transcription factor for ion channels and transporters in these tissues (Burglin and Ruvkun 2001; Armstrong and Chamberlin 2010). Null mutants die early in the first larval stage. The knockdown animals were viable, but had very short canals with large fluid-filled cysts. The effect of *ceh-6* knockdown could reflect the expected lower transcription levels of canal transporters and the excretory aquaporin *aqp-8* (Mah *et al.* 2007), as well as the possibility of direct effects on other known or novel *exc* genes.

Knockdowns in *egal-1*, *mop-25.2*, F41E7.1 (*exc-11*), and T05D4.3 (*exc-12*) exhibited small-to-medium-sized cysts (Figure 2C-F). In these animals, cystic regions of the lumen often appear to consist of a series of hollow spherical domains, which may be connected or separate from each other along the lumen length.

#### Large vesicles and swollen cytoplasm:

The largest group of knockdown animals showed generally normal-diameter lumen surrounded by accumulation of larger vesicles outside the lumen and swollen canal cytoplasm (Figures 3, 4). In some cases, the swelling occurs primarily at the distal tip, and appears to be caused by accumulation of a convoluted lumen folded back on itself, while in other knockdowns this swelling reflect accumulation of a large number of vesicles at the end of the lumen. A combination of these structures also appears in many animals. Reflecting the variable effects of RNAi knockdown, some animals knocked down in ceh-37 (in which knockdowns generally caused less extreme effects, see below), and in a gene mentioned above, *mop25.2*, sometimes showed a highly convoluted lumen primarily at the distal tip (Figure 3A, 3B), possibly reflecting weaker knockdown than in other examples where the entire lumen was affected. Knockdown of other genes, *best-3* and *gck-3*, showed a similar effect (Figure 3C, 3D). Knockdowns of *fbxa-183* (Figure 3E) show clear and dramatic accumulation of large vesicles at the swelling at the tip of the lumen. A few knockdowns of *gst-28* gave similar but less reproducible effects (Fig. S1).

In other cases, the swollen cytoplasm and presence of vesicles was distributed along the length of the canals (Figure 4). Knockdown of the gene K11D12.9 (to be referred to as *exc-14*) exhibited an extraordinary increase of vesicles in the cytoplasm of the canal, terminating in a large irregular swelling at the terminus of the canal (Figure 4A, 4A’). In these animals, the short lumen of the canal appears relatively normal in diameter, but is surrounded by cytoplasm that puffs out at the basal side of the cell. GFP labeling of the cytoplasm showed a thick layer of fluorescence surrounding the lumen suggestive of normal canaliculi excluding larger vesicles, and which is surrounded by a cytoplasm packed with vesicles of variable size.

Some animals knocked down for the *fbxa-183* gene (Figure 4B) also exhibited large vesicles surrounding an irregular lumen, (in addition to those *fbxa-183* knockdowns with large cysts discussed above). Knockdowns of T08H10.1 (to be referred to as *exc-15*) similarly exhibited large numbers of these variable-sized vesicles in the canal cytoplasm (Figure 4C). The enlarged lumen was also apparent in DIC micrographs of animals of the “million-mutation” strain VC20239 that contains a substitution mutation of this gene (Figure 4C’).

Finally, knockdown of two other genes, C03G6.5 (*exc-13*) and *cyk-1* (Figure 4D, 4E) caused the appearance of large cysts or vesicles appearing at the basal surface of the canals in just a few seemingly random spots along the length of the canals, as well as at the distal tips of the canals. A few of the “million-mutation” animals containing a point mutation in *exc-13* also showed an enlarged shortened canal lumen, although large cysts along the canal length were not visible (Figure 4D’, Table S2). Similar but less reproducible effects were seen for knockdowns of H09G03.1 and in a few of the “million-mutation” animals containing substitution mutations of the H09G03.1 gene (Fig. S1, Table S2).

A very narrow canal “tail” completely lacking a visible lumen often extends substantially past the end of the lumenated portion of the canal in several of the knockdown animals (marked by arrows in Figure 3A, 3C, 3D, S1B). This tail follows the path of wild-type canal growth, and in rare instances even reaches the normal endpoint of the canal. In wild-type animals, the lumen and tip of the canal grow together and reach the same endpoint (Buechner *et al.* 1999), with a widening suggestive of a growth cone at the tip of the growing canal in the embryo and L1 stage (Fujita *et al.* 2003). The tip of the canal is enriched in the formin EXC-6, which mediates interactions between microtubules and actin filaments and may mediate connections between the canal tip and end of the lumen (Shaye and Greenwald 2015). The results here are consistent with the idea that canal lumens grow and extend separately from the growing basal surface that guides cytoplasmic outgrowth (Kolotuev *et al.* 2013).

####  Periodic cytoplasmic swellings:

Knockdown of some genes caused animals to exhibit shorter canals with periodic swellings of the cytoplasm (Figure 5) rather than luminal distension. These swellings, also called “beads” or “pearls,” are commonly seen in wild-type animals with rapidly growing canals at the L1 stage, and in animals under osmotic stress (Kolotuev *et al.* 2013). These sites were hypothesized in that study to be locations of addition of membrane to allow the canal to continue to grow together with the animal. The knockdown animals here were measured in young adulthood, after the period of rapid growth, and so presence of these beads may reflect osmotic stress caused by partial loss of the encoded proteins. Knockdown of the *inx-12* or *inx-13* genes (Figure 5A, 5B), which encode innexins highly expressed in the canals (and in the adjacent CAN neurons), gave rise to these structures. Innexins form the gap junctions of invertebrates (Hall 2017), and the excretory canals are rich in these proteins along the basal surface, where they connect the canal cytoplasm to the overlaying hypodermis (Nelson *et al.* 1983). Null mutants in either of these two genes results in early larval rod-like swollen lethality consistent with excretory cell malfunction (Altun *et al.* 2009). The knockdown phenotype here further suggests that these proteins regulate balancing of ionic content to allow normal canal growth.

A similar phenotype is seen in animals knocked down for *ceh-37* or *mxt-1*, and, with lower statistical confidence, for knockdown of *dhhc-2* (Figure 5C, 5D, Fig. S1). The *dhhc-2* knockdown animals exhibited somewhat enlarged dark spots consistent with the presence of many variably enlarged vesicles within the beads, and the beads themselves show more variable size and placement than for the other knockdowns in this class.

#### Variability of canal phenotypes:

Some knockdowns of *ceh-37* resulted in regular beads along the canal length (Figure 5C), while others resulted in a swollen lumen (Figure 3A). Expression of *ceh-37* is itself regulated by CEH-6 (Burglin and Ruvkun 2001), knockdown of which caused large cysts (Figure 2A). The ultimate canal phenotype in all of these treated animals likely represents the degree of gene knockdown as well as the nature of the specific protein affected by knockdown. This hypothesis is supported from the results seen from RNAi-knockdown of the *vha-5* gene (Figure 6). This gene encodes a protein of the membrane-bound V_0_ subunit of the vacuolar ATPase (Oka *et al.* 2001). Mutations of this gene are lethal, and a point mutation led to strong whorls of labeled VHA-5 at the apical surface (Liégeois *et al.* 2006). Here, knockdown of *vha-5* resulted in a wide range of canal phenotypes in different animals (Figure 6). Some animals exhibited beads surrounding a normal-diameter lumen (Figure 6A), similar to animals under osmotic stress, as in Figure 5. Other animals showed small septate cysts in the canal lumen, but the canal lumen overall was of near-normal diameter, and the basal surface had mostly minor irregularities (Figure 6B), similar to animals knocked down for *exc-15* (Figure 4C). Other *vha-5* knockdown animals exhibited a similar luminal phenotype, but also showed large vesicles within a highly irregular-shaped cytoplasm (Figure 6C), similar to animals impaired in *exc-13* or *cyk-1* expression (Figure 4D, E). Finally, the most extremely affected *vha-5* knockdown animals (Figure 6D) showed cysts throughout the lumen, a swollen terminus to the lumen, and a range of variable-sized vesicles or cysts that pack the entire swollen cytoplasm of the canals.

### Other Phenotypes

**Figure 7:**
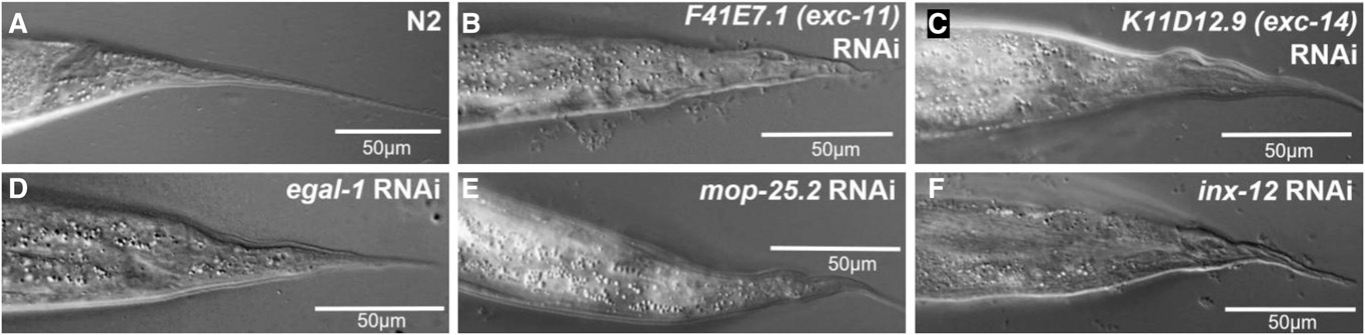
Knockdown of some *exc* genes causes tailspike defect. DIC images of the narrow tail spike of adult hermaphrodite wild-type animal (A) and of adult mutants exhibiting RNAi knockdown for genes. Knockdowns of: (B) *F41E7.1* (*exc-11*); (C) *K11D12.9* (*exc-14*); (D) *egal-1*; (E) *mop-25.2*; (F) *inx-12*.

While the focus of this RNAi screen centered on excretory canal morphology, a few other phenotypes were noted, including occasional effects on gonadal shape, fertility, and viability. In many *exc* mutants, the shape of the normally smooth hermaphrodite tail spike (Figure 7A) is affected (Buechner *et al.* 1999), and similar strong results were reproducibly observed here for multiple RNAi knockdowns (Figure 7B-F). In addition to the knockdowns shown (for genes *exc-11*, *exc-14*, *egal-1*, *mop-25.2*, and *inx-12*), tail spike defects were also seen in animals knocked down in genes encoding homeobox protein CEH-6, vacuolar ATPase component VHA-5, sedoheptulose kinase EXC-10, aldo-keto reductase EXC-15, and innexin INX-13 (data not shown). The tail spike is formed from the interaction of hypodermal tissue hyp10 with a syncytium of two other hypodermal cells that later undergo cell death (Sulston *et al.* 1983); it remains to be determined what features this structure has in common with the canals that require the same proteins.

### Suppressors of the Exc-5 Phenotype

Finally, the RNAi screen was also carried out in animals carrying mutations in various *exc* genes, to try to find genes that interacted to form more severe phenotypes. Previous interactions have found, for example, that *exc-3*; *exc-7* double mutants have a more severe canal phenotype than does either mutant alone (Buechner *et al.* 1999), and similar synergetic effects are seen for *exc-5*; *exc-6* double mutants (Shaye and Greenwald 2016). No such effects were detected in this screen, unfortunately. We conclude that the variability of knockdown strengths and resultant wide range of canal length in the starting strains prevented easy identification of severely affected animals at the initial screening step, so that such enhancer mutations could not be easily identified.

**Figure 8:**
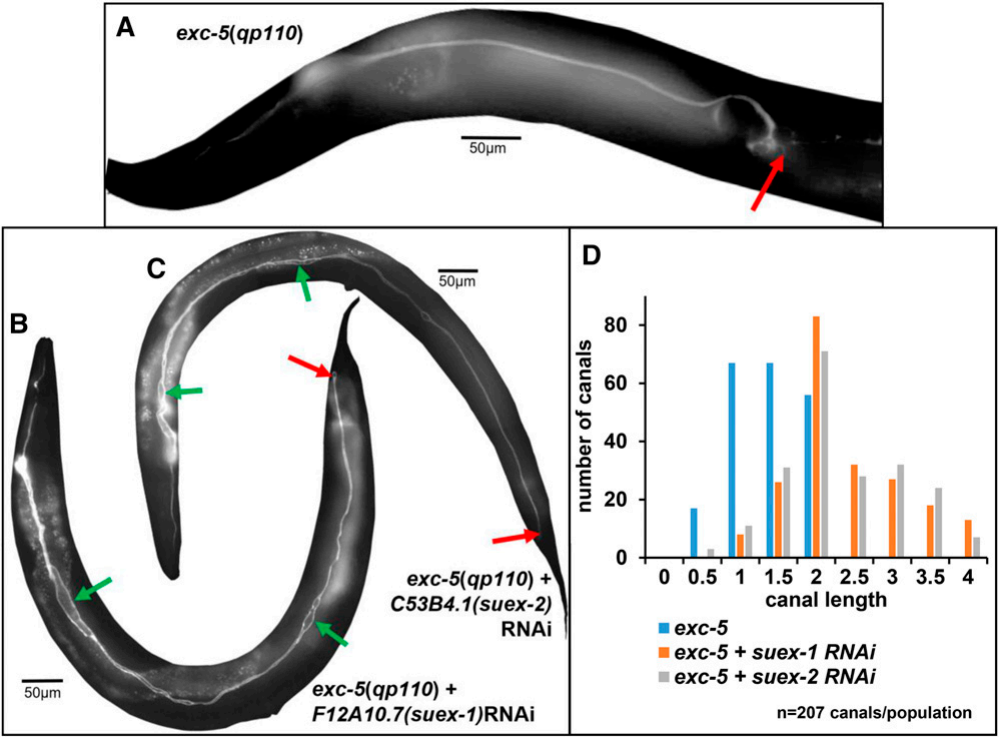
Knockdown of two genes suppresses the Exc-5 phenotype. (A-C) GFP fluorescence of canals in BK545 (*exc-5*(*rh232*) null mutants) with RNAi-sensitized background and GFP expressed in canal cytoplasm (A) and of BK545 animals showing strong suppression when knocked down for (B) F12A10.7 (*suex-1*), or (C) C53B4.1 (*suex-2*). Exc-5 phenotype includes very short normal-diameter canals terminating in large cysts. Red arrows indicate termination of canals. Green arrows indicate areas of slight swelling of Suex canal lumen in both knockdowns. (D) Measurement of effect of *suex* suppression via feeding RNAi on canal length. Canals from *exc-5* mutant and mutants with *suex* knockdown were measured according to scale in Fig. 1A. Average canal length: *exc-5*(*rh232*): 1.4, *exc-5*(*rh232*); *suex-1*(*RNAi*): 2.3, *exc-5*(*rh232*); *suex-2*(*RNAi*): 2.3. N = 207 for each genotype. Analysis via 3x2 Fisher’s Exact Test (see Materials and Methods) show differences from wild-type canal length that are highly significant: *P* of 9.0x10^−17^ for *suex-1*, 1.7x10^−13^ for *suex-2*.

Knockdown of two genes, however, caused an unexpected phenotype: the restoration of near-wild-type phenotype from strongly cystic homozygous *exc-5*(*rh232*) animals (Figure 8) carrying a large deletion of almost all of the *exc-5* gene (Suzuki *et al.* 2001). *exc-5* encodes a guanine exchange factor (GEF) specific for CDC-42 (Gao *et al.* 2001; Suzuki *et al.* 2001), and mutants are defective in transport from early endosomes to recycling endosomes (Mattingly and Buechner 2011). EXC-5 is homologous to four human FGD proteins, including two that are implicated in Aarskog-Scott Syndrome (Facio-Genital Dysplasia) and Charcot-Tooth-Marie Syndrome Type 4H, respectively (Gao *et al.* 2001; Delague *et al.* 2007; Horn *et al.* 2012). The latter disease affects outgrowth of the single-celled tubular Schwann cells during rapid growth, and identification of mutations in suppressor genes therefore has the potential to increase understanding of this disease.

*exc-5* null mutants are characterized by large fluid-filled cysts at the terminus of both anterior and posterior canals (Figure 8A). Knockdown RNAi of these suppressor genes, both by feeding and by direct dsRNA microinjection, yielded a large number of progeny exhibiting near-normal canal phenotypes (Figure 8B, 8C), with canal length extending near-full-length (Figure 8D). We will refer to this phenotype as Suex, for SUppressor of EXcretory defects. In SUEX canals, no obvious septate cysts are evident, although parts of the canal lumen were slightly widened (Figure 8B, 8C).

## Discussion

### Proteins Encoded by the Knockdown Genes

Several types of protein appear repeatedly as products of the genes with knockdown effects on the canals (Table 3). Significantly, knockdown of some proteins with similar functions appear in different phenotypic classes. This observation again suggests that the difference between large and smaller cysts, and between cyst formation and vesicle accumulation, may reflect the relative expression level of the proteins involved rather than fundamentally different processes involved in preventing cyst formation or vesicle accumulation within the canals.

#### Transcriptional and post-transcriptional regulation:

Since this screen focused on genes preferentially expressed within the canals, it is not surprising that transcriptional factors affecting canal expression were among the proteins identified. As noted above, the POU-domain transcription factor CEH-6 (Figure 2A) regulates transcription of many genes in the canal (Burglin and Ruvkun 2001; Armstrong and Chamberlin 2010), including the well-conserved OTX Homeobox gene *ceh-37* (Figure 3A), expression of which is restricted to the excretory canals in adults (Lanjuin *et al.* 2003; Hench *et al.* 2015). In addition, the EGAL-1 (Figure 2C) exonuclease is homologous to *Drosophila* Egalitarian, which degrades RNA (Fridolfsson *et al.* 2010), and the F-box protein FBXA-183 (Figure 3E, 4C) facilitates targeting substrates for E3 ubiquitinase-mediated destruction (Kipreos and Pagano 2000). EXC-14 (K11D12.9) (Figure 4A) encodes a protein containing a RING finger domain at the C-terminus, with BLASTP homology to the human CGRRF ubiquitin ligases that regulates ER stress (Kaneko *et al.* 2016). MXT-1 (Figure 5D) binds both to mRNA and to eukaryotic initiation factor 4E to regulate translation rate (Peter *et al.* 2015).

#### Cytoskeletal Regulators:

Several cytoskeletal proteins are critical for canal structure. The ACT-5 actin, and EXC-2 and IFB-1 intermediate filaments are expressed predominantly in the canals and intestines, both tissues with a thick actin-based terminal web that restricts apical expansion (Macqueen *et al.* 2005; Kolotuev *et al.* 2013; Al-Hashimi *et al.* 2018). Mutants in the SMA-1 β_H_-spectrin or the ERM-1 ezrin/radixin/moesin that bind actin to the apical membrane allow dilation of the canal into very large cysts (Mckeown *et al.* 1998; Göbel *et al.* 2004; Khan *et al.* 2013). The present screen confirmed the role of the CYK-1 *Diaphanous* formin homolog (Severson *et al.* 2002) in canal morphogenesis; this protein was previously found to interact with the EXC-6 formin to regulate the EXC-5 guanine exchange factor (Mattingly and Buechner 2011; Shaye and Greenwald 2016). RNAi knockdown of *cyk-1* here (Figure 4E) produced a stronger phenotype (shorter canals with large cysts on the basal side) than seen in the temperature-sensitive mutant used by the Greenwald laboratory, but not as strong an effect as was seen in double mutants of *cyk-1*(*ts*) with *exc-6* null mutants (Shaye and Greenwald 2016).

#### Ion transport:

Vacuolar ATPase is an important ion pump that generates proton gradients in excretory tissues from *Paramecium* to human (Wassmer *et al.* 2008), it is strongly expressed in the canals (Oka *et al.* 2001), and knockdown of vacuolar ATPase genes affects morphology of multiple tissues, including the canals (Liégeois *et al.* 2006; Hahn-Windgassen and Van gilst 2009), so it was expected to see strong effects from knockdown of the *vha-5* gene within the canals (Figure 6). In addition, INX-12 and INX-13 (Figure 5A, 5B) encode innexins, key components of invertebrate gap junctions (Hall 2017). The excretory canals are rich in these proteins along the basal surface, where they connect the canal cytoplasm to the overlaying hypodermis (Nelson *et al.* 1983), as well as in the adjacent CAN neurons that are postulated to control excretory canal function (Manser and Wood 1990; Altun *et al.* 2009). Null mutants in either of these two genes results in early larval rod-like swollen lethality consistent with excretory system malfunction (Altun *et al.* 2009).

This screen found three additional genes that encode proteins likely to regulate canal cell ion content: *best-3*, exc-*11*, and the suppressor gene *suex-2*. BEST-3 (Figure 3C) is homologous to the mammalian chloride channel bestrophin, essential for Ca^++^ signaling in muscles, neurons, and eyes, and defective in retinal diseases (Strauß *et al.* 2014).

EXC-11 (F41E7.1) (Figure 2E) and SUEX-2 (C53B4.1) (Figure 8C) are both previously unstudied proteins with homology to members of the ubiquitous SLC (SoLute Carrier) proteins found in a wide range of animals, including humans. EXC-11 appears to be a member of the SLC9 family (SLC9B subgroup) of Na^+^/H^+^ antiporters found in plasma membrane and endosomes, and might be expected to exchange the acidic protons of canalicular vesicles for sodium (Fuster and Alexander 2014), in order to increase luminal osmolarity and draw excess body water into the canals to be excreted. SUEX-2 is a transporter that has been implicated in gonadal distal tip cell migration in a previous RNAi screen (Cram *et al.* 2006). Its closest homolog SLC22A1 encodes a 12-tm-domain integral membrane protein transporting organic cations (Nigam 2018) and expressed in the human liver and small intestine. The effect of knocking down this transporter implies that ionic milieu or lipid composition affects transport of vesicles mediated by EXC-5 signaling, but future work will be needed to determine the role that this transporter exerts on ionic content, and possibly on endosomal recycling in the developing excretory canal cell.

#### Regulation of excretory cell vesicle transport:

In addition to SUEX-2, SUEX-1 (F12A10.7) (Figure 8B) also presumably regulates transport of endosomes within the excretory canals, which is the process disrupted through impairment of the EXC-5 guanine exchange factor. The small SUEX-1 protein (113 amino acids) is unique to *C. elegans*, expressed in the excretory canal cell and in some neural subtypes, with homology to genes in only a few other *Caenorhabditis* species. The C-terminal half of the protein contains a number of repeats of tri- and tetra-peptides GGY and GGGY.

Two other genes were identified here that likely affect transport of canal vesicles. MOP-25.2 (homolog of MOuse embryo scaffolding Protein 25) (Figure 3B) acts as a scaffolding for endocytic recycling, and reduced the number of vesicles expressing the recycling endosome marker RAB-11 in a recent canal mutant screen (Lant *et al.* 2018). That screen searched for interactors of the protein CCM-3 (for Cerebral Cavernous Malformations), a homolog of a mammalian protein that regulates vascular integrity in the brain. Mutants in *ccm-3* and other components of the CCM complex also show strong canal defects, though not as severe as those found for loss or reduction of MOP-25.2 function (Table 2).

The STE20-related kinase GCK-3 was also identified in our screen (Figure 3D). GCK-3 is homologous to cell-volume-regulating kinases (Falin *et al.* 2009; Gagnon and Delpire 2012), and phosphorylates to inactivate the voltage-gated intracellular chloride channel CLH-3 found in the excretory cell as well as in the egg-laying HSN neurons and enteric muscles (Denton *et al.* 2005; Miyazaki *et al.* 2012). Interestingly, in *Drosophila*, MOP-25.2 has also recently been found to regulate ion transport in the Malpighian Tubule GCK-3 through activation of WNK kinase signaling (Sun *et al.* 2018). The present knockdown results are consistent with these other findings and with the above observations and conclusions from the Derry laboratory screen (Lant *et al.* 2018) that MOP-25.2 likely acts in multiple pathways to regulate excretory cell shape.

#### Enzymatic activities:

Two surprising genes found in this screen encode proteins with homology to enzymes involved in lipid and sugar metabolism. EXC-15 (T08H10.1) (Figure 4C) has strong homology to aldo-keto-reductase family 1 member B10, a human intestinal protein that may detoxify aromatic aldehydes and ketones, and is overexpressed in tumor tissues (Barski *et al.* 2008). In a previous RNAi screen, knockdown of the *C. elegans* gene slowed the defecation rate by about 20%, possibly through effects on mitochondrial stress (Liu *et al.* 2012). *exc-10* (T25C8.1) (Figure 2B) encodes a carbohydrate kinase (with homology to sedoheptulose kinase, an enzyme of the glycolytic pentose phosphate pathway) of unknown function in nematodes. The human homolog SHPK has been linked to a lysosomal storage disease (Phornphutkul *et al.* 2001; Wamelink *et al.* 2008).

#### Novel proteins:

EXC-12 (T05D4.3) (Figure 2F), EXC-13 (C03G6.5) (Figure 4D), and SUEX-1 (F12A10.7) (Figure 8B) proteins have no known function and no obvious homology except to other nematode proteins. SUEX-1 even appears to be unique to the Caenorhabditids. While the majority of proteins with strong effects on canal structure have close homologs in a wide range of eukaryotes, the discovery of these proteins implies that the universal structure of unicellular tubes can be modified or regulated for the specific phylum and family.

### Source of Swollen Vesicles

As described above, many of the mutant phenotypes involve the accumulation of large vesicles in various regions of the excretory canals, and the source of these vesicles is an unanswered question from this study. Several types of vesicles are visible in electron micrographs of the excretory canals (Nelson *et al.* 1983). The canal lumen is surrounded by myriad strings of small vesicles that are each encased in a thick electron-dense coat that includes the vacuolar ATPase (Kolotuev *et al.* 2013). These vesicles remain separate from the lumen, or can attach to each other and the lumen to form small canaliculi that allow pumping of protons into the lumen. In electron micrographs of many of the previously described *exc* mutants for which electron micrographs were taken (Buechner *et al.* 1999), the number and placement of canalicular vesicles around sections of the canal lumen varies greatly, but when present, the canalicular vesicles in these mutants, as for wild-type animals, have a diameter of about 80-110 nm.

Larger vesicles visible in the light microscope are marked by various fluorescently tagged Rab proteins (Tong and Buechner 2008) that have been used to delineate the complicated processes of secretion and endocytosis in cells of *C. elegans* by labeling early, recycling, and late endosomes (Sato *et al.* 2014). Labeled vesicles derived from these endosomes do not have an obvious thick protein coat, are larger than canalicular vesicles, and in electron microscopic images, are predominantly found outside the region of canalicular vesicles. In *exc-1*, *exc-5*, and *exc-9* mutants, the early endosomes are greatly enlarged (Tong and Buechner 2008; 2011; Grussendorf *et al.* 2016). In the present study, knockdown of *exc-14* clearly results in large vesicles that are excluded from a sub-apical domain surrounding the lumen (Figure 4A), as would be expected if canalicular vesicles are intact as in wild-type animals.

On the other hand, the vacuolar ATPase proteins, including VHA-5, are clearly highly enriched on canalicular vesicles, and knockdown of this gene results in the wide range of vesicular defects described in this study. Mutants affecting the small GTPase RAL-1 that regulates canalicular vesical fusion appear to result in fewer but larger vesicles (Armenti *et al.* 2014). Mutants defective in CCM-3/PDCD10, a protein that resides in several complexes that regulate mammalian vascular integrity (preventing Cerebral Cavernous Malformations), also show variation in vesicle size, with larger vesicles with a thick coat that may contain vacuolar ATPase located farther from the luminal surface than are smaller vesicles closer to the apical surface (Lant *et al.* 2015; Pal *et al.* 2017). Significantly, mutants in *mop-25.2* have recently been shown to affect the location of CCM-3 in the excretory canals (Lant *et al.* 2018).

While we postulate that the large vesicles in these knockdown animals are generally of endosomal origin, final identification of the origin of canalicular vesicles as endosomally derived, apical-membrane-derived, or both, therefore awaits detailed study of mutants in these genes.

## Conclusion

This RNAi screen was successful at identifying 18 genes (10 not implicated before) needed to form a normal lumen of the long excretory canals of *C. elegans*. These genes encode transcription and translation factors, innexins and other channels, and proteins involved in subcellular trafficking, among others. While these processes have been implicated previously in canal tubulogenesis, these proteins represent new actors that could provide insights into how these cellular processes are integrated in single-cell tubulogenesis. Several proteins, such as sedoheptulose kinase, could introduce insight into previously unidentified processes involved in canal morphogenesis. Finally, two additional genes were identified as suppressors of *exc-5* mutation; determining the function of these suppressor proteins has the potential to increase understanding of the role of FGD protein function during normal and in disease state single-cell tubulogenesis.
